# Supplementary material for: Precise Epigenetic Analysis Using Targeted Bisulfite Genomic Sequencing Distinguishes FSHD1, FSHD2, and Healthy Subjects
Source: Diagnostics (Basel). 2021 Aug 13;11(8):1469. doi: 10.3390/diagnostics11081469 (PMC8393475; doi:10.3390/diagnostics11081469)
Supplement: Supplementary file 1 [file diagnostics-11-01469-s001.zip › diagnostics-1308497-supplementary.pdf]

Supplementary Materials

Precise Epigenetic Analysis Using Targeted Bisulfite Genomic Sequencing Distinguishes FSHD1, FSHD2, and Healthy Subjects

Table S1. Oligonucleotide primers used for BS-PCR (5′→3′).

BIS-3′	F:	GTTTTGTTGGAGGAGTTTTAGGA
	R:	CTAAACCTAAAAAACAAAAATCCA **
BIS-5′	F:	AAATATGTAGGGAAGGGTCTAAGTT
	R:	CTTAAATATACCAAACCCTCTCTCC **
BIS-Mid	F:	ATTTATGAAGGGGTGGAGTTT
	R:	ATAACCTAAACCAACCGTTCTCTA **
BSSA-1438	F:	GTTTTGTTGGAGGAGTTTTAGGA
BSSA-3742	R:	AACATTCAACCAAAATTTCACRAAA
BSSA-3626	R:	AACAAAAATATACTTTTAACCRCCAAAAA
BSSL	F:	TTATTTATGAAGGGGTGGAGTTTGTT
BSSX-167	F:	TTTTGGGTTGGGTGGAGATTTT
BSSX-475	F:	TTAGGAGGGAGGGAGGTAG
BSSX-1036	R:	AACACCRTACCRAACTTACACCCTT

** The reverse primers were published as the Watson strand sequence in Gaillard et al., 2014. These show the corrected Crick strand sequences with 5′→3′orientation.

Table S2. Epigenetic analysis using the Jones et al. BSSL method for 4qAL alleles.

Subject	Genetic Diagnosis	Haplotype	DRA	BSSL Avg	BSSL Q1	BSSL Q2	BSSL Q3	Epigenetic Diagnosis
C-01	Healthy	4A161/4A161L	>48kb	77.8	73.3	76.7	85	Healthy
C-02	Healthy	4A161/4A161L	>48kb	81.8	76.7	80	86.45	Healthy

We have found that the BSSL assay for 4A161L allele shows much higher levels of methylation associated with FSHD1 than the BSSA assay. Therefore, we use Q1 methylation levels to determine FSHD or not FSHD.

Table S3. Epigenetic analysis using the Jones et al. BSSA method on subjects with a 4A161/4A161 haplotype.

Subject	BSSA Avg	BSSA Q1	BSSA Q2	BSSA Q3	BSSX Avg	BSSX Q1	BSSX Q2	BSSX Q3	Epigenetic Diagnosis
C-05	52.8	42.9	56.25	64.3	56.8	34.2	64.4	78	Healthy
C-06	64.6	59.8	69.1	73.2	55.3	46.65	52.5	65.25	Healthy
C-07	58.2	52.25	55.4	65.15	48.1	31.6	43.25	61.85	Healthy
C-08	52.1	42.9	51.8	60.7	37.5	20.03	35.05	44.95	Healthy
F1-01	30.9	0.9	28.6	58.95	37.6	22	33.9	58.95	FSHD1
F1-06	14.8	8.9	28.55	62.5	64.7	68.5	73	75.05	FSHD1
F1-07	31.9	1.8	27.95	68.75	64.2	49.15	69.5	78	FSHD1
F1-08	32.9	8	14.3	60.35	63.8	61	67.8	79.7	FSHD1

This analysis utilizes the relevant quartile (yellow boxes), which corresponds to Q1 for those with two 4A161 alleles and Q2 for those with one 4A161 allele, to determine the methylation status.

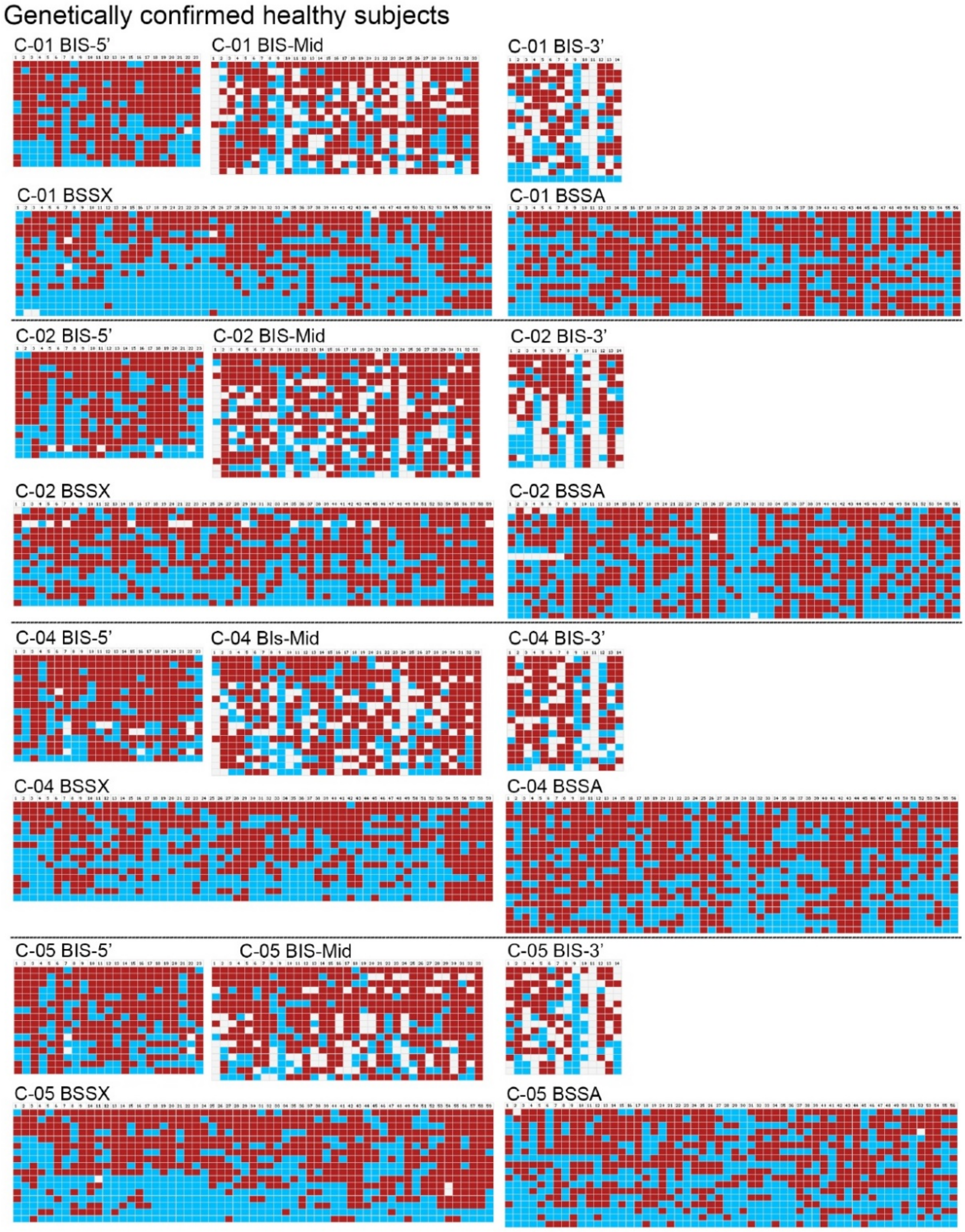

Figure S1. BSS analysis comparing healthy epigenetic signatures. BS-converted gDNAs from four genetically confirmed healthy subjects were analyzed using the BIS-5′, BIS-Mid, BIS-3′, BSSX, and BSSA assays. Blue boxes indicate unmethylated CpGs, red boxes indicate methylated CpGs, and white boxes indicate no CpG where expected.

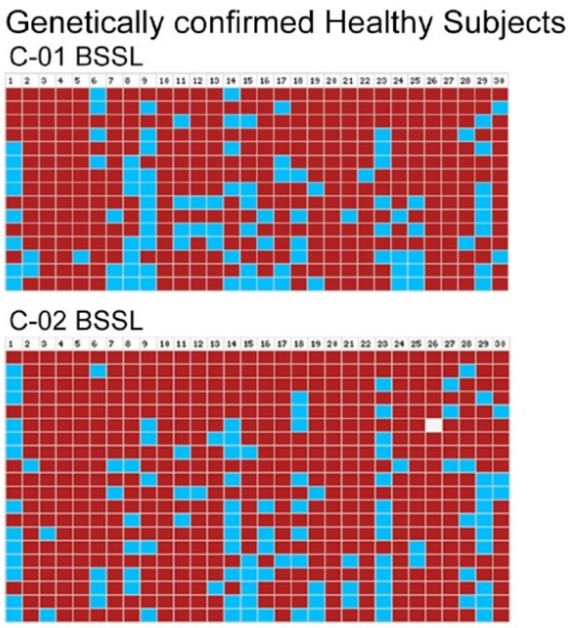

Figure S2. BSS analysis using the BSSL assay for two healthy subjects. BS-converted gDNAs from two healthy controls with a 4A161/4161L haplotype were analyzed using the BSSL assay in addition to the BSSA assay. The results of the BSSL assay show the 4A161L allele is hypermethylated in both subjects. When combined with the results of the BSSA, we conclude these subjects are healthy. Blue boxes indicate unmethylated CpGs, red boxes indicate methylated CpGs, and white boxes indicate no CpG where expected.

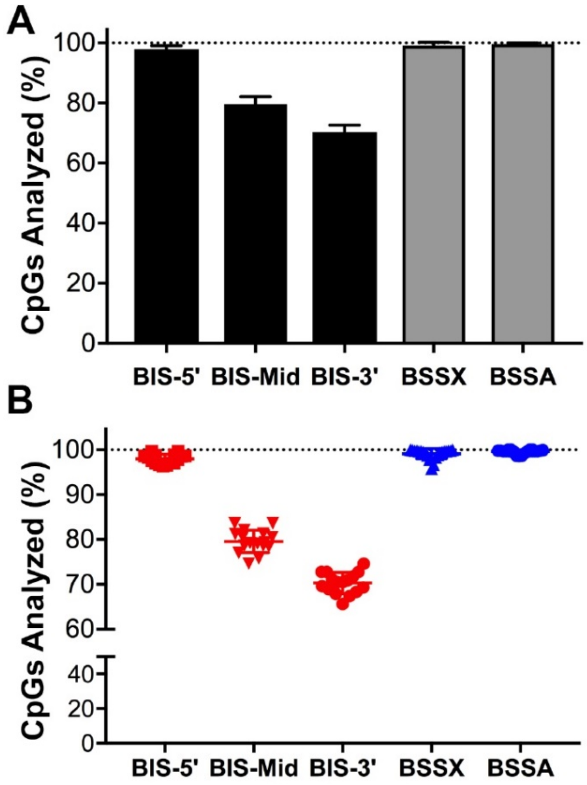

Figure S3. Graphs plotting the percentage of CpGs analyzed compared with those in the reference sequence for each BSS assay are indicative of the specificity of each amplicon for the 4q35 and 10q26 D4Z4 arrays. (**A**) Bar graphs for the total analyzed CpGs as a percentage of total predicted CpGs in each of the reference sequences when amplified from the 4q35/10q26 D4Z4 arrays. (**B**)Each individual assay, by subject, is plotted as in (**A**). BSSA assay, n = 22, BSSX assay, n = 22, BIS assays, n = 16.

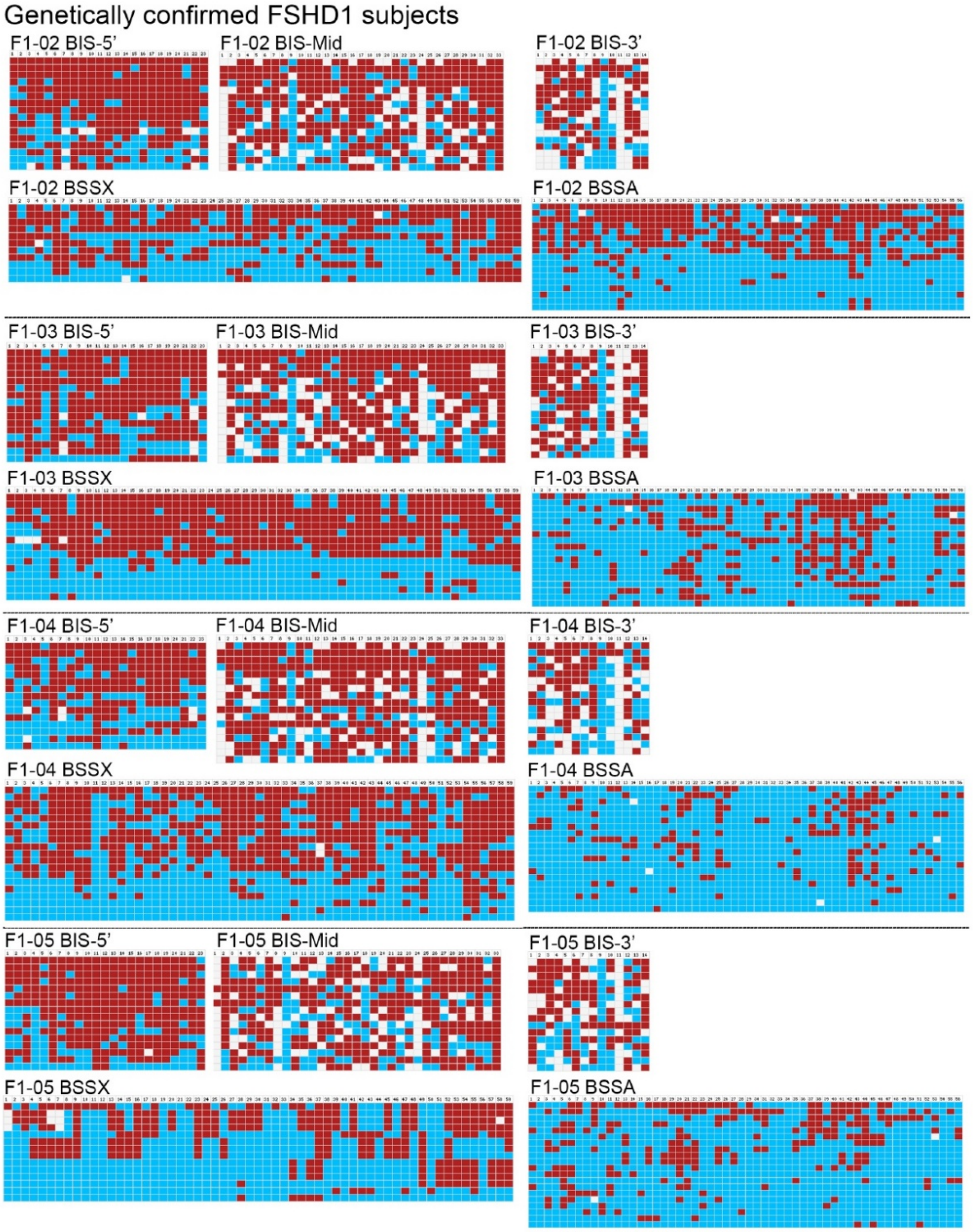

Figure S4. BSS analysis comparing FSHD1 epigenetic signatures. BS-converted gDNAs from four genetically confirmed FSHD1 subjects were analyzed using BIS-5′, BIS-Mid, BIS-3′, BSSX, and BSSA assays. Blue boxes indicate unmethylated CpGs, red boxes indicate methylated CpGs, and white boxes indicate no CpG where expected.

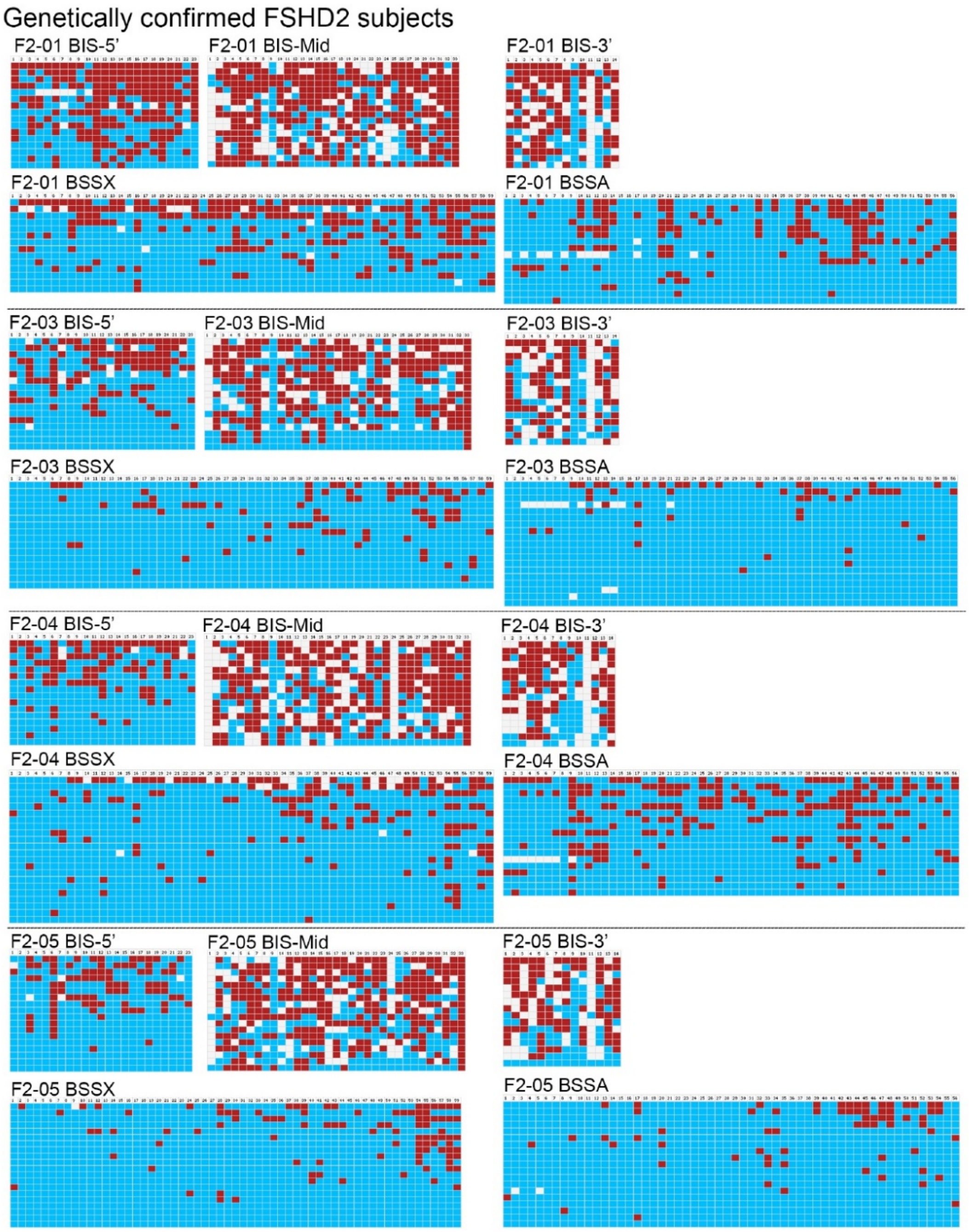

Figure S5. BSS analysis comparing FSHD2 epigenetic signatures. BS-converted gDNAs from four genetically confirmed FSHD2 subjects were analyzed using BIS-5′, BIS-Mid, BIS-3′, BSSX, and BSSA assays. Blue boxes indicate unmethylated CpGs, red boxes indicate methylated CpGs, and white boxes indicate no CpG where expected.

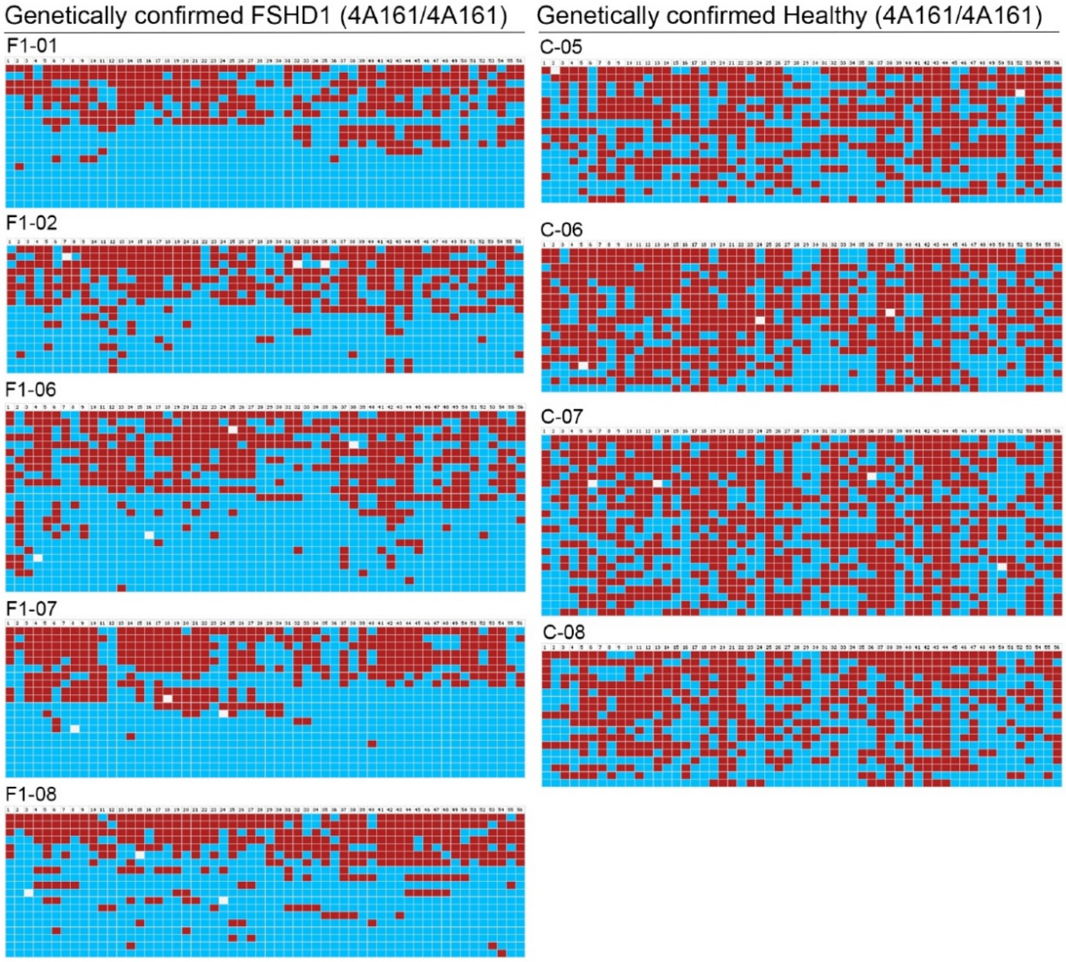

Figure S6. BSSA analysis of FSHD1 and healthy controls with 4A161/4A161 haplotype finds no bias for the methylation state. BS-converted gDNAs from five genetically confirmed FSHD1 subjects and four genetically confirmed healthy subjects with a haplotype of 4A161/4A161 were analyzed using the BSSA assay. Blue boxes indicate unmethylated CpGs, red boxes indicate methylated CpGs, and white boxes indicate no CpG where expected. Two clear and roughly equal populations, one hypomethylated and one hypermethylated, are evident in the FSHD1 subjects. Similarly, the healthy control subjects do not show any preference for less methylated sequences.
